# Unique combination of hyaluronic acid and amino acids in the management of patients with a wide range of moderate‐to‐severe chronic wounds: Evidence from international clinical practice

**DOI:** 10.1111/iwj.14630

**Published:** 2024-02-23

**Authors:** Emre Özker, Arkadiusz Krakowiecki, Roberto Cassino, Carla Pezzuto, Paul Chadwick, Marco Romanelli

**Affiliations:** ^1^ Altınbaş University Faculty of Medicine, Head of Cardiovascular Surgery Acıbadem Health Group Wound Clinics Istanbul Turkey; ^2^ Chronic Wound Clinic Podos Warsaw Poland; ^3^ “Residenze Heliopolis” Korian Nursing Home Milan Italy; ^4^ Lecturer at the Master of Vulnology University of Turin Turin Italy; ^5^ Department of Plastic Surgery Burn Unit and Skin Bank Turin Italy; ^6^ Tissue Viability Birmingham City University School of Health Sciences Birmingham UK; ^7^ Division of Dermatology, Department of Clinical and Experimental Medicine University of Pisa Pisa Italy

**Keywords:** amino acids, case reports, consensus, hyaluronic acid, wounds

## Abstract

The availability of new products and strategies to manage wounds has taken a quantum leap in recent years. Healthcare professionals now have an extensive range of products to choose from, but while positive this also raises dilemmas in real‐world clinical practice to decide on the most appropriate treatment for a given patient. Clinical trials confirm the effectiveness of the unique combination of hyaluronic acid and amino acids (Vulnamin®) in a range of wounds, but are these results replicated in real‐world clinical practice? International experts presented their clinical experience with the use of the combination in difficult‐to‐treat wounds. The objective was to reach a consensus on how and when to use the unique combination products to provide a cost‐effective, convenient option, in all healthcare settings that improves QoL for patients and their carers.

## 1 INTRODUCTION

Evidence from clinical trials confirm the effectiveness of the unique combination of hyaluronic acid and amino acids in a range of wounds^1^, ^2^, ^3^, ^4^, ^5^, ^6^ but is this replicated in real‐world clinical practice? Members of the expert panel presented their clinical experience with the combination product range. Case reports presented are from the following European countries—Italy, Poland, Turkey and the United Kingdom. Data collection was in accordance with the principles of Good Clinical Practice and the Declaration of Helsinki, and all the participating patients provided written informed consent.

## 2 CASE NOTES (AUTHOR EO)

### 2.1 Case 1: A combination of hyaluronic acid and amino acids powder in Buerger disease

A 46‐year‐old female smoker with Buerger disease was initially referred to our clinic in 2021 for wound treatment but was lost to follow‐up. She returned 12 months later—in the interim, she attended another clinic where angioplasty was performed but failed and she was treated with adipose tissue stem cells, cilostazol, aspirin and pain medications with topical antibiotic cream—the wound on her right foot healed but ulcers on her left toes worsened.

She had wounds (not infected) with eschars on her first and second toes—first toe: mild, non‐purulent discharge, nail detached, Wagner Grade 3 with osteitis; second toe: Wagner Grade 2 penetrating to joint capsule.

**FIGURE 1:**
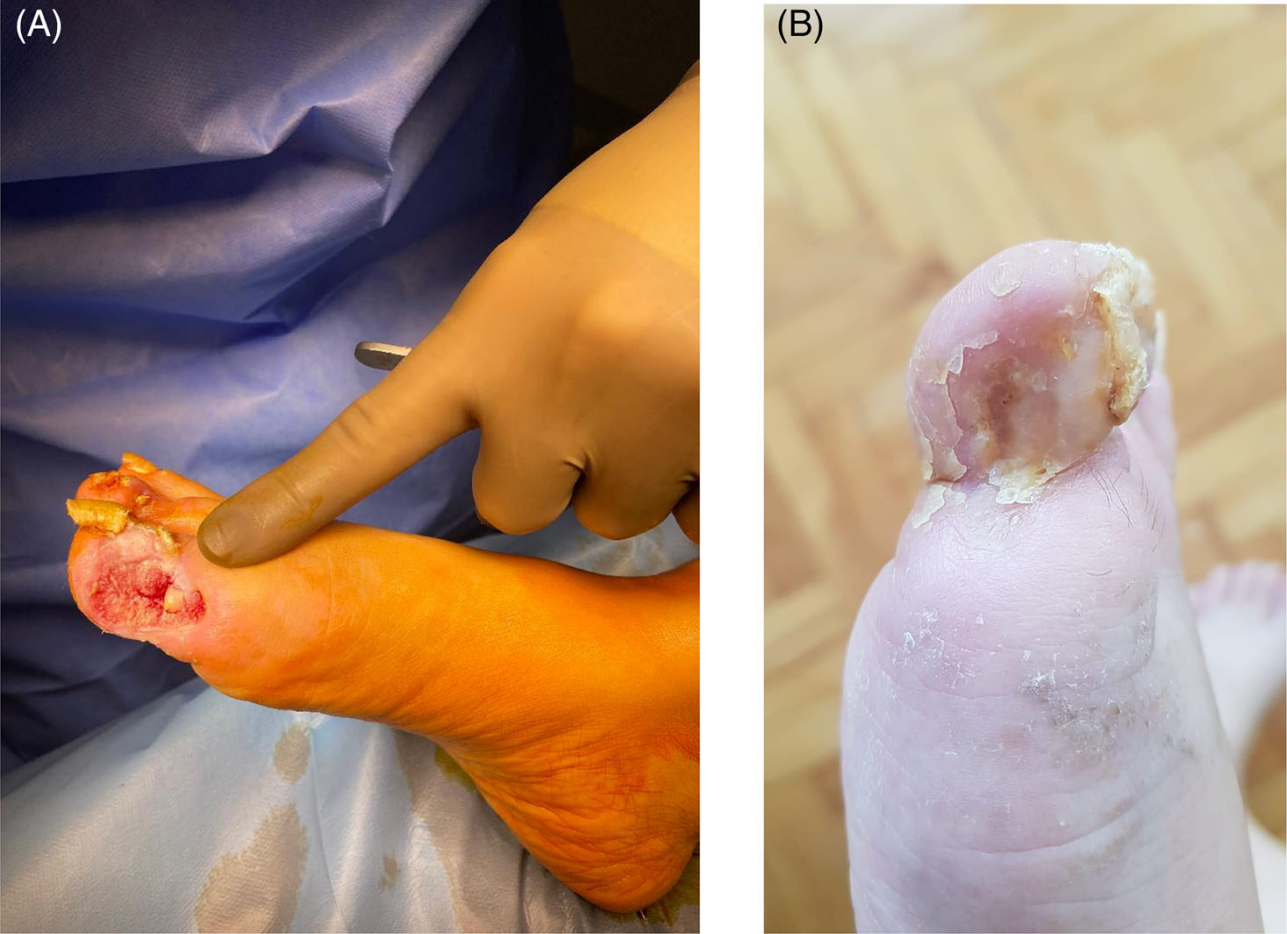
Case 1: (A) Before and (B) after treatment with unique combination of hyaluronic acid and amino acids (powder).

Treatment started with debridement and eschar removal and proximal phalanx uncovered, and partial bone resection was performed. A combination of hyaluronic acid and amino acids powder and silver foam dressing (PolyMem silver) were applied every other day (except weekends) for a total of 13 applications over 1 month. Pain significantly decreased after the first week, and repeat debridement was not necessary. The bone became fully covered with granulation tissue after the third application, and rapid granulation and concomitant epithelization were observed. A unique combination of hyaluronic acid and amino acids treatment allowed fast healing which led to a reduction in pain (VAS decreased from 9 to 3) with no side effects reported. At the end of treatment, the wound was closed totally (Figure 1A,B).

**Take‐away messages**

- Despite absence of any highly sophisticated scaffold use, very fast and successful coverage of open bone structure.
- Despite very difficult clinical scenario (vasculitis‐Buerger disease with unsuccessful angioplasty, anaemia and open bone) and undertreatment (no repetitive debridement), the clinical course was flawless.
- No need for other advanced wound products, the combination powder provides adequate moist environment.
- Wound completely closed at the end of treatment.

### 2.2 Case 2: A combination of hyaluronic acid and amino acids powder in diabetic foot

A 51‐year‐old male attended our out‐patient clinic with a non‐healing wound following toe amputation (1 month earlier in another clinic) and a wound in second toe manifest after the amputation procedure. The patient had refused anterior foot amputation in the previous clinic. He was an ex‐smoker, with a 20‐year history of type 2 diabetes, kidney transplant and metabolic surgery in 2020, retinal detachment in 2010. He was treated with mycophenolate mofetil, tacrolimus, prednisolone, clopidogrel, aspirin and repaglinide.

After amputation he had a non‐infected, Wagner Grade 1 wound (bed covered with granulation tissue with mild exudation) and mild pain when pressure exerted on the flexor hallucis longus muscle. The lateral side of second toe was covered with eschar, the medial side had a Wagner Grade 2 wound, and the toe was colder than the foot.

Pedal pulses on his left foot were not palpable, biphasic flow in anterior tibial artery, monophasic flow in posterior tibial artery, ankle/brachial index (ABI) left foot: 1.42 (ABI right: 0.86—no wound in right leg). He had anaemia (Hb:12.3 g/dL) and was HbA1c: 5.7%. He underwent surgery, his second toe was amputated, and he returned to his home country. He returned to our clinic and was re‐operated on, the flexor hallucis longus trajectory was opened and debrided, the first metatarsal bone tip was debrided, and he again returned home. The patient subsequently sent photos to our clinic of a detached suture line—some stitches removed, and negative pressure wound therapy (NPWT) was started. Wound treatment continued with collagen laminin‐based dermal matrix combined with resveratrol microparticles (Dermalix) together with foam dressings.

**FIGURE 2:**
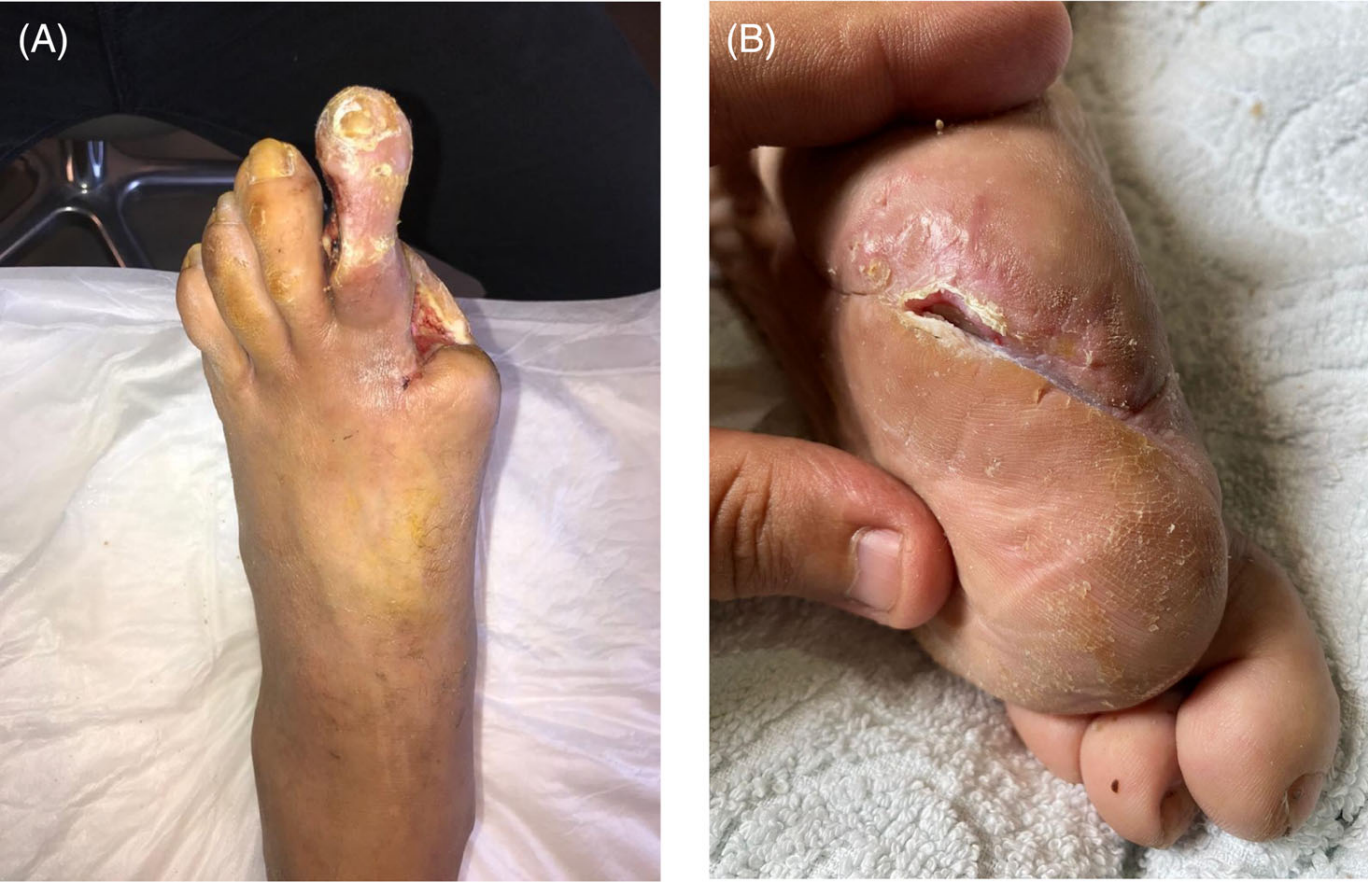
Case 2: (A) Before and (B) after treatment with unique combination of hyaluronic acid and amino acids (powder).

The patient was called back to our clinic for surgery—second metatarsal head and some metatarsal bone from the remaining first metatarsal bone was resected. Stitches dehisced again 1‐week post‐operation—the patient returned to clinic and stitches were renewed. Partial necrosis began after the renewal of the stitches at the first week. The patient called back for debridement (18 March–22 April 2022), and treatment with the combination powder and foam dressing (coloplast biatain) once every other day was started immediately. The wound depth was 1.5 cm, and the bone was partially exposed. The patient responded very well to treatment, and a total of 18 applications of the combination powder were carried out and the wound almost closed in 32 days (98% in 4 weeks) (Figure 2A,B).

**Take‐away messages**

- The combination powder (hyaluronic acid and amino acids) worked well against systemic factors that delay wound healing: patient was receiving immunosuppressant and steroid therapy.
- Patient had chronic disease‐related anaemia and heavily diseased arteries (high ABI indicating advanced media sclerosis).
- Wound area had undergone four surgical procedures, three resulted in poor healing, indicating problematic wound healing mechanisms.
- Despite very difficult clinical scenario, wound healing after the combination powder was fast and aesthetically acceptable.
- Very fast and successful coverage of open bone structure.
- No need for additional sophisticated wound products—any product that provides moist wound treatment and thermal insulation is sufficient.

## 3 CASE NOTES (AUTHOR AK)

### 3.1 Case 3: A combination of hyaluronic acid and amino acids in venous leg ulcer management

The patient was a 72‐year‐old female with a venous leg ulcer; she was a heavy smoker and had extensive oedema and a history of repeated unsuccessful angioplasty and chemotherapy for breast cancer. At a clinic visit in early 2020, we recommended she stop smoking before therapy could begin (ABI 0.28, C‐reactive protein [CRP 210 mg/L]). In June 2020, she had partial angioplasty (external iliac artery only EIA; control ABI 0.4) and lymphatic drainage, walking training, haemoglobin spray, first degree compression. In January 2021, she had an unsuccessful angioplasty and was not suitable for hyperbaric oxygen therapy (ABI 0.33, TcpO2 28 mmHg), and her ulcer worsened but there was no infection.

**FIGURE 3:**
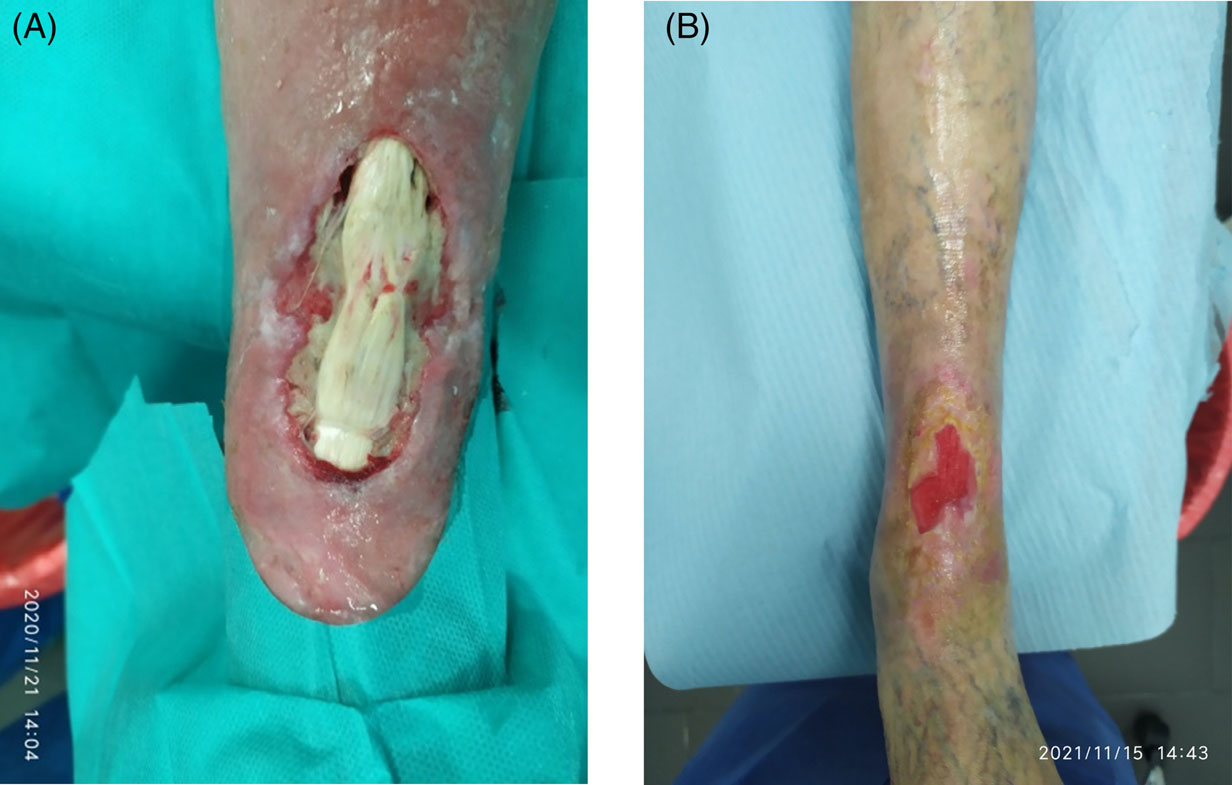
Case 3: Treatment with unique combination of hyaluronic acid and amino acids (powder) (A) started November 2020 and (B) following 1 year of treatment, November 2021.

The combination powder started in November 2020 and continued until November 2021 when the wound was successfully treated (Figure 3A,B).

### 3.2 Case 4: A combination of hyaluronic acid and amino acids in diabetic foot ulcer management

**FIGURE 4:**
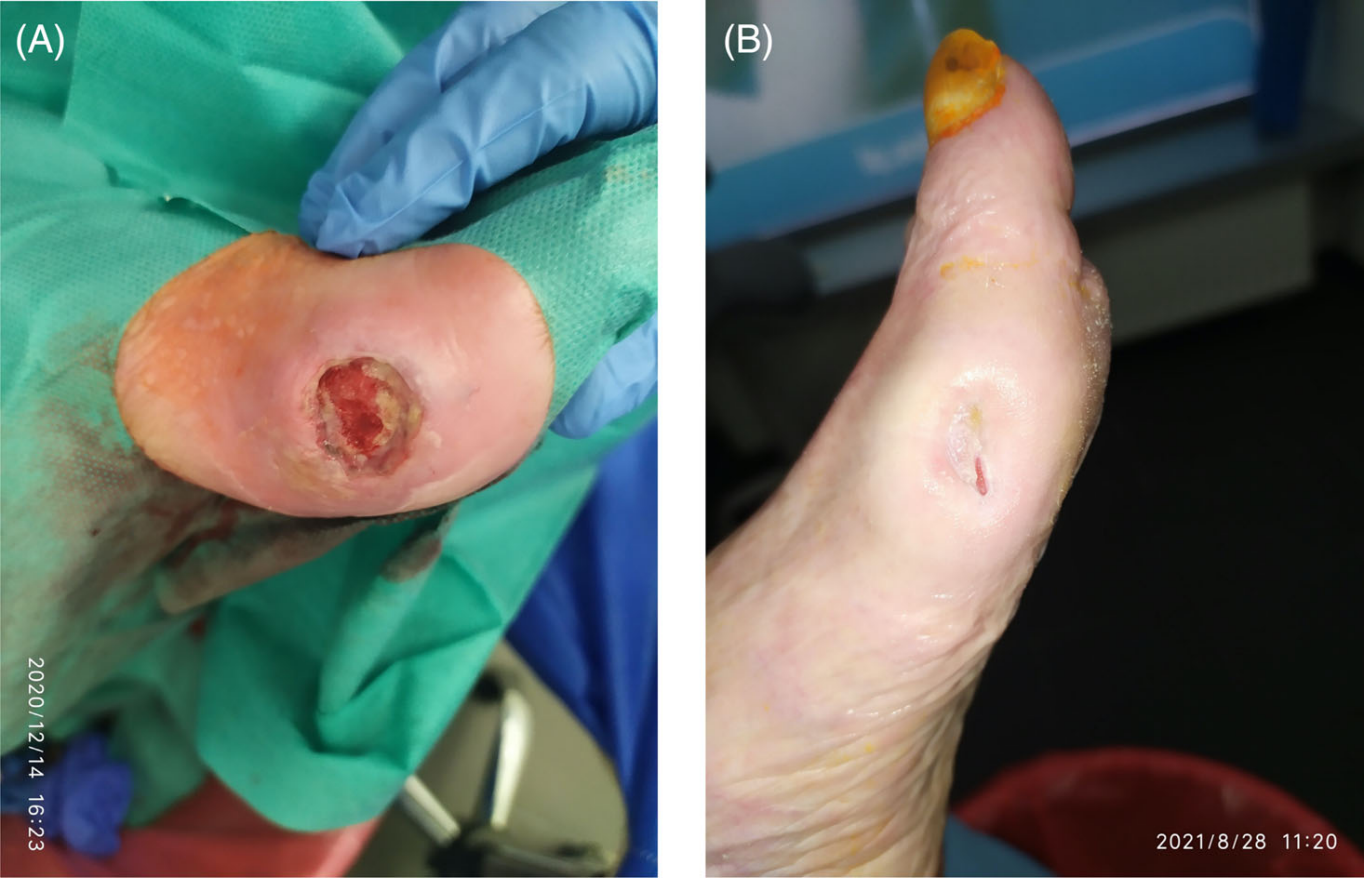
Case 4: Diabetic foot ulcer (A) before and (B) after treatment with unique combination of hyaluronic acid and amino acids for 8 months.

Ninety‐four‐year‐old female presented with diabetic foot ulcer due to long‐term type 2 diabetes (Hb A1c 6.7, ABI 0,23 TBI 0,0). She had occlusion of superficial femoral artery, anterior tibial artery and posterior tibial artery. Angioplasty was unsuccessful. She was managed with walking training, electric stimulation of muscles and the expanded bone was removed. She was treated with a combination of hyaluronic acid and amino acids in powder format, and her ulcer was completely healed following 8 months of treatment (Figure 4A,B).

### 3.3 Case 5: A combination of hyaluronic acid and amino acids in powder format in venous leg ulcer management

**FIGURE 5:**
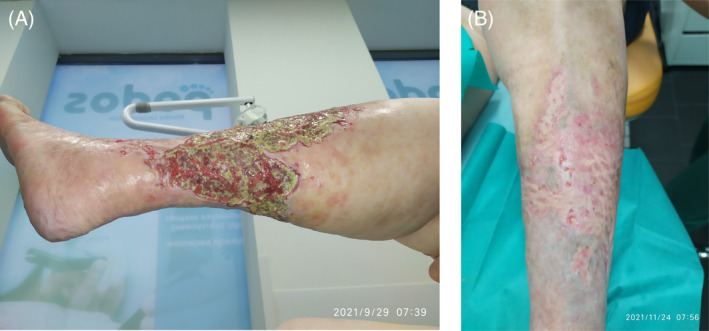
Case 5: Ulcer (A) before and (B) after treatment with unique combination of hyaluronic acid and amino acids for 2 months.

A 73‐year‐old female presented with recurrent leg ulcers since 2009—her previous history included chronic leukaemia (in remission), right hemicolectomies in 2011 and proctocolectomies in 2015. She presented at our clinic in late 2021; she had NPWT in combination with compression bandaging (100 mL exudate per day) and skin grafts. The wound successfully treated with the combination of hyaluronic acid and amino acids in powder format over a 2‐month period (Figure 5A,B).

### 3.4 Case 6: A combination of hyaluronic acid and amino acids in diabetic foot ulcer management

Our patient was a 32‐year‐old female with long‐standing type 2 diabetes, hypertension, amputation of the right hallux in 2018, phlegmon of the foot and amputation of third left toe in late 2021. She has been undergoing haemodialysis since late 2021. She was admitted to our clinic for amputation—debridement, necrotic tissue removed and antibiotics started. An X‐ray showed no base infection.

**FIGURE 6:**
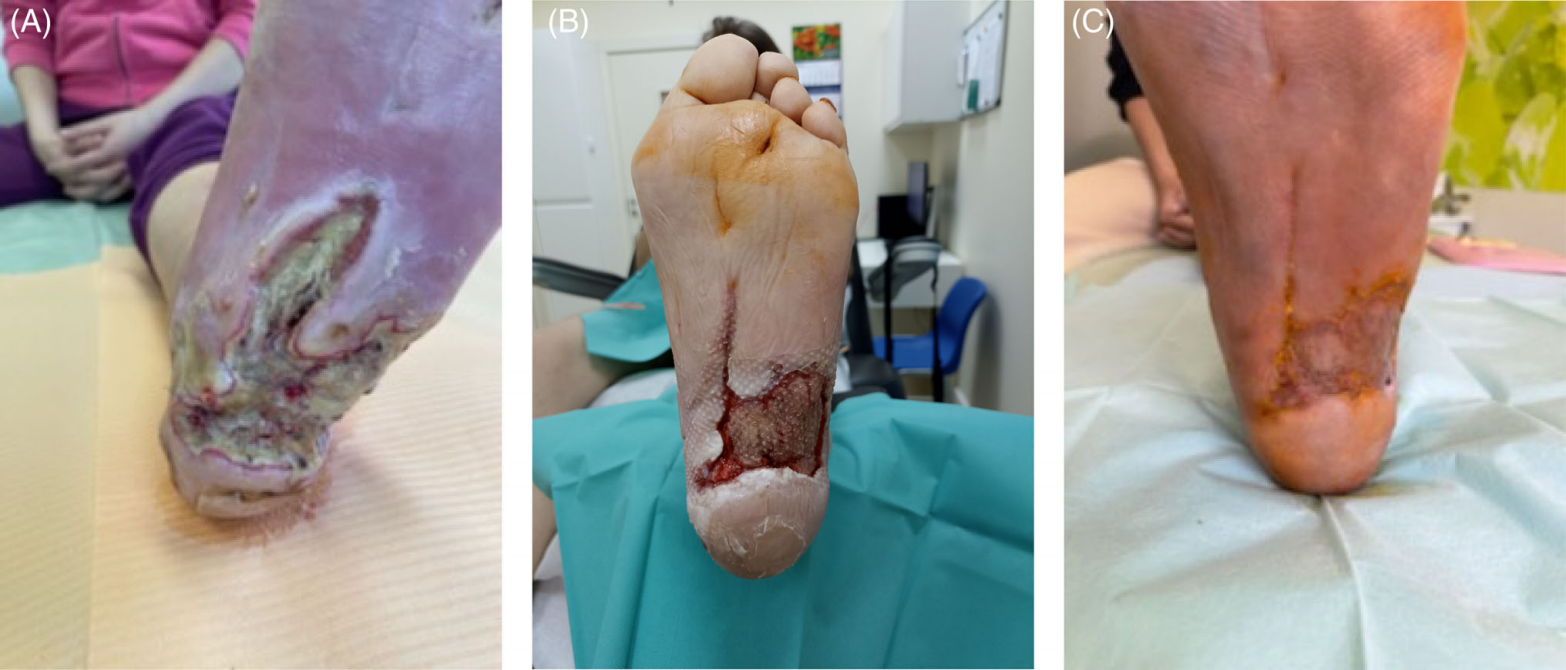
Case 6: Diabetic foot ulcer (A) on 09 March 2022 before treatment with unique combination of hyaluronic acid and amino acids, (B) on 25 April 2022 and (C) on complete healing in January 2023.

The patient had a deep pocket (3 cm) on her foot which was filled with the combination powder (foam not used). There was a rapid (March to April 2022) almost complete closure of the wound. The patient made a good recovery with complete healing on follow‐up in January 2023 (Figure 6A–C).

### 3.5 Case 7: A combination of hyaluronic acid and amino acids in diabetic foot ulcer management

A female 38‐year‐old patient presented with a 10‐year history of type 2 diabetes and Hashimoto's disease. She had a severe infection in her left foot for several months before presenting at our clinic—she was hospitalized, and investigations showed normal ABI and a palpable pulse.

**FIGURE 7:**
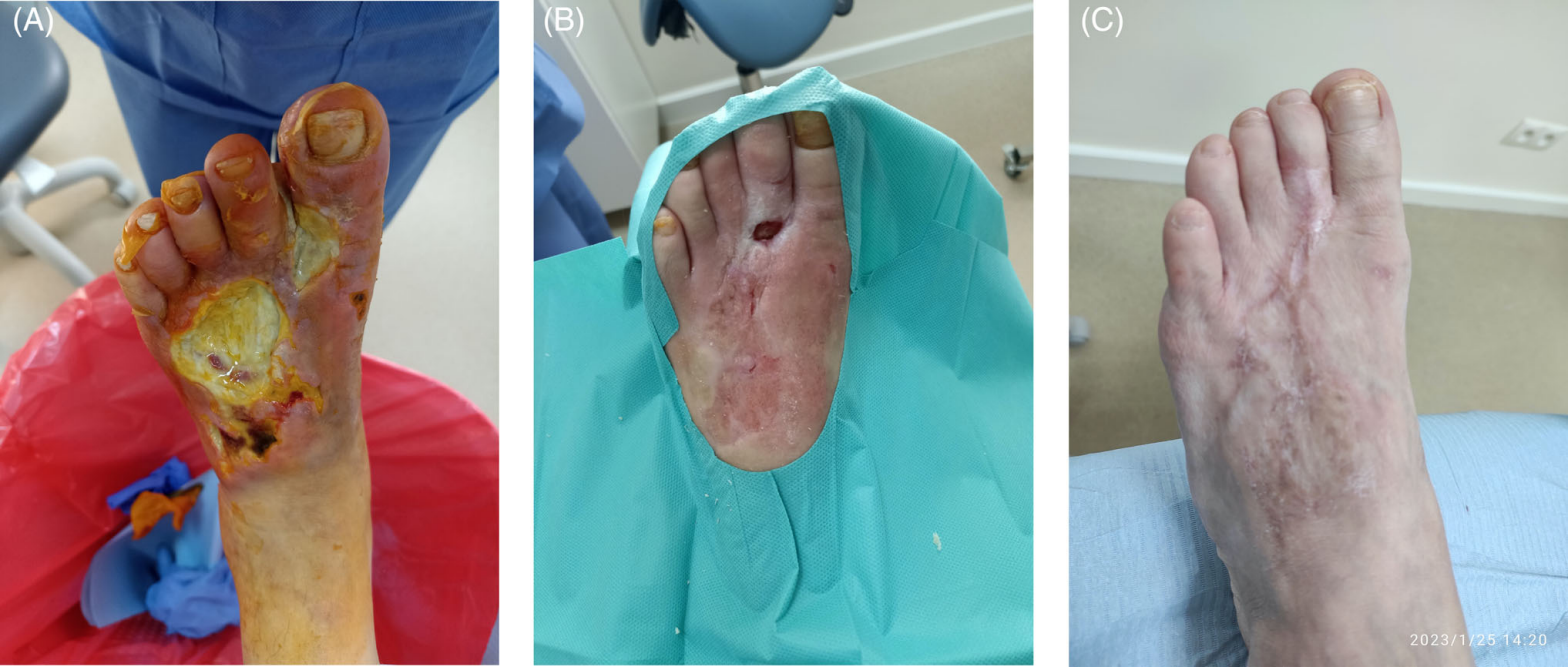
Case 7: Diabetic foot ulcer (A) on 20 October 2021 before treatment with unique combination of hyaluronic acid and amino acids, (B) on 21 April 2022 and (C) on 25 January 2023 on complete healing.

We decided to first use maggot therapy, but she was in severe pain and 1 month of NPWT did not produce any improvement. We then decided to begin treatment with the combination powder and after 1 month, we started to see a major improvement. She had a skin graft in November 2021, and in April 2022, her wound had almost completely closed, and she could use normal shoes with special insoles. On follow‐up in early 2023, the wound was completely healed (Figure 7A–C).

**Take away messages**

A combination of hyaluronic acid and amino acids in powder format

- Improved granulations and epithelialization.
- Improved deep pocket closing.
- Reduction of failed split‐thickness skin grafting.

## 4 CASE NOTES (AUTHOR RC)

### 4.1 Case 8: A combination of hyaluronic acid and amino acids in post‐phlebitis syndrome management

An 87‐year‐old woman presented with post‐phlebitis syndrome (prior deep vein thrombosis) complicated by phlebolymphedema, severe chronic obstructive pneumopathic disease (receiving oxygen therapy, 4 L/min for a minimum of 20 h a day) and chronic kidney failure (stage V—patient refused haemodialysis).

The skin on the posterior section of her leg was severely compromised with major transcutaneous transudation due to sudden worsening of oedema. The patient was treated with hyaluronate gauzes, but she had severe pain, increased maceration and general worsening.

**FIGURE 8:**
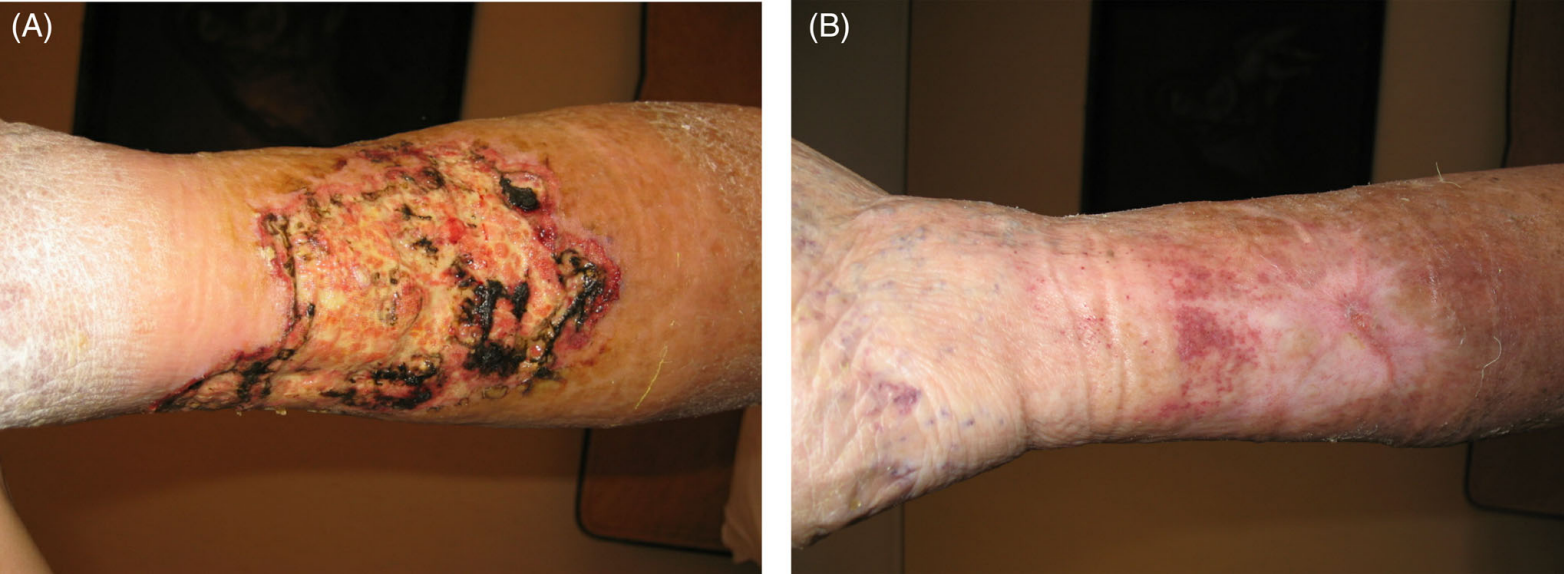
Case 8: Post‐phlebitis syndrome (A) before and (B) after 130 days of therapy with unique combination of hyaluronic acid and amino acids (powder).

We decided to introduce the combination of hyaluronic acid and amino acids in cream format three times a week with petrolatum gauzes used as secondary dressings. After 130 days of the combination cream treatment, there was a complete resolution and, importantly, there was a well hydrated and smooth scar, and the patient was pain‐free (Figure 8A,B).

### 4.2 Case 9: A combination of hyaluronic acid and amino acids gel format in the management of Buerger disease

A 91‐year‐old female smoker with Buerger disease, developed ischemic gangrene of 4th and 5th toes of her right foot resulting in the need for amputation. After about 3 weeks, surgical dehiscence observed with necrotic tissue and exposure of the extensor tendon of the third toe. She was treated with povidoiodine ointment and gauzes, but her condition worsened.

**FIGURE 9:**
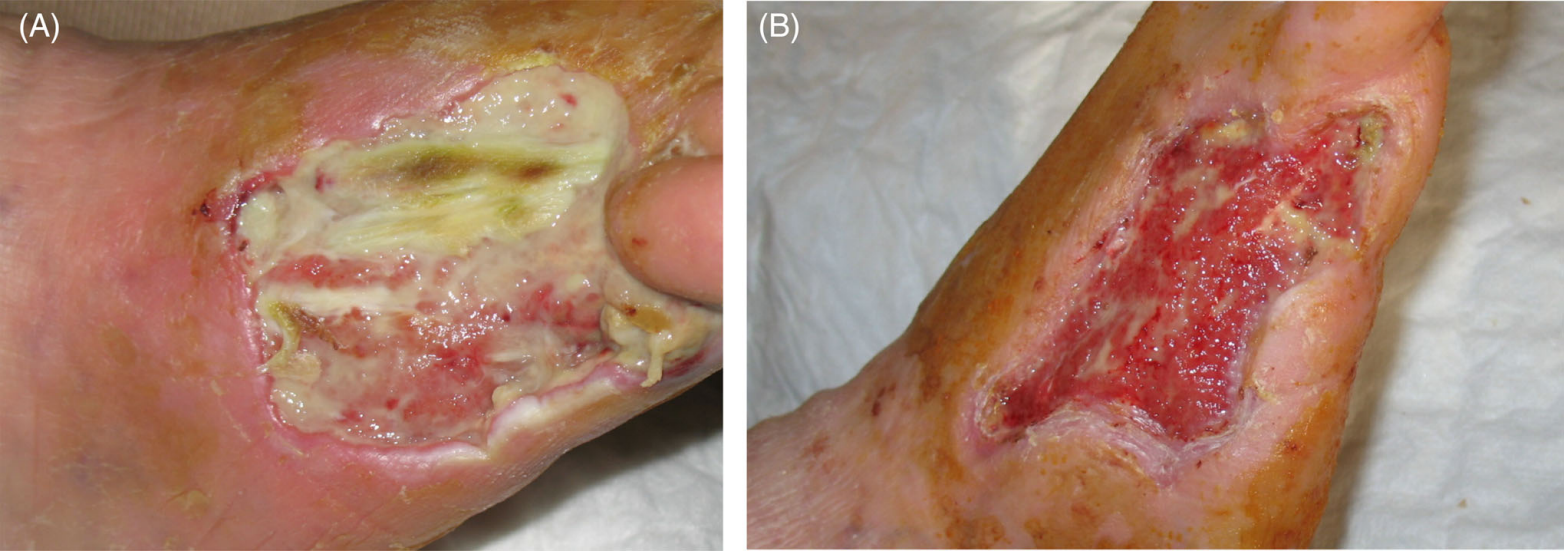
Case 9: Buerger disease (A) before and (B) after 5 weeks of therapy with unique combination of hyaluronic acid and amino acids (gel) (3 applications/week).

We started treatment with the combination gel for 5 weeks with 3 applications/week, using povidoiodine gauzes as secondary medication. Treatment with the combination gel resulted in a rapid improvement. There was quick debridement and epithelialization with granulating tissue. She did not experience any adverse reaction/allergies or pain (Figure 9A,B).

### 4.3 Case 10: A combination of hyaluronic acid and amino acids in cream format in the management of lichenification

A 72‐year‐old woman with cognitive impairment as a result of childhood meningitis developed incontinence‐associated dermatitis and subsequently lichenification in the groin area which intermittently became complicated with fissures, often bloody and painful. She was treated unsuccessfully with zinc oxide cream.

**FIGURE 10:**
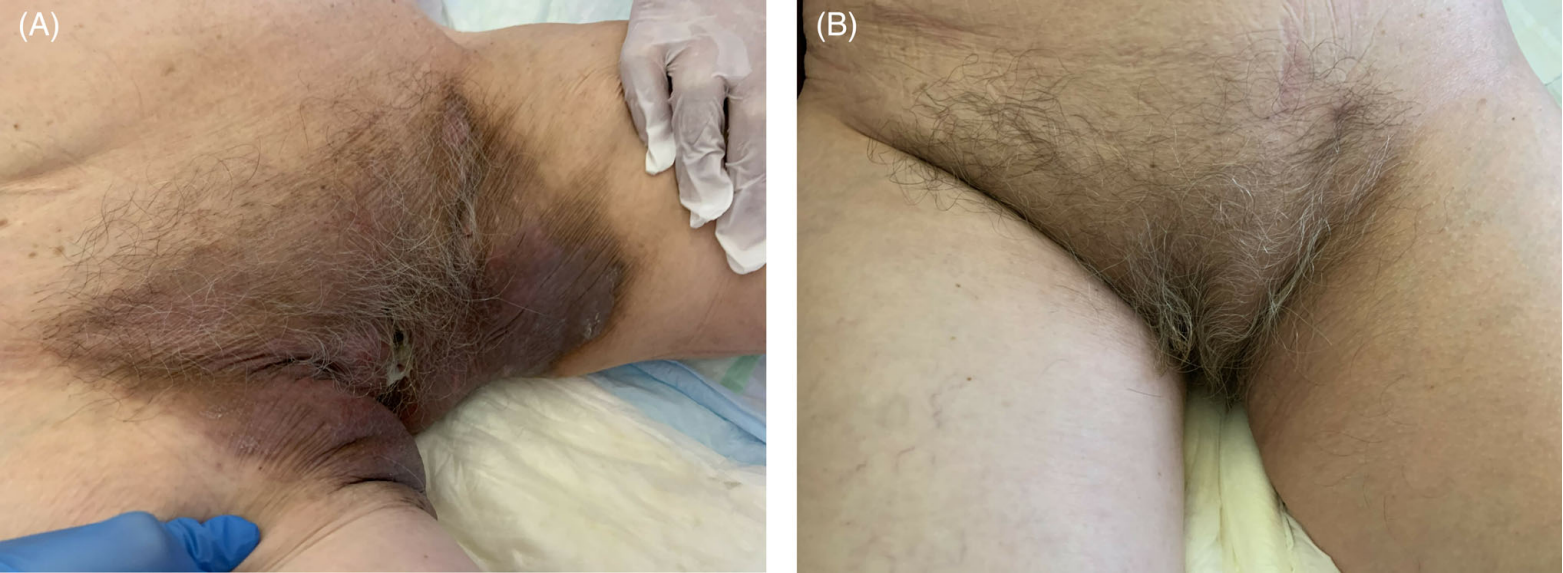
Case 10: Lichenification (A) before and (B) after treatment with unique combination of hyaluronic acid and amino acids (cream) for 4 weeks (2 applications per day) with a 90‐day follow‐up.

The combination cream twice a day was tried as a last resort for 4 weeks with two applications per day with a 90‐day follow‐up. Her condition improved dramatically, and lichenification disappeared after 3 months. We consider this a new off‐label indication for the management of lichenification and also scar treatment. There was rapid epithelialization, complete recovery of the skin (well hydrated and elastic), no pain and no adverse reactions/allergies (Figure 10A,B).

### 4.4 Case 11: A combination of hyaluronic acid and amino acids in spray format to prevent skin lesions

A 102‐year‐old diabetic patient presented with immobilization syndrome resulting from stroke, chronic atrial fibrillation, diabetic cardiomyopathy, stage IV chronic kidney failure and faecal and urinary incontinence and as a result she developed multiple pressure injuries.

**FIGURE 11:**
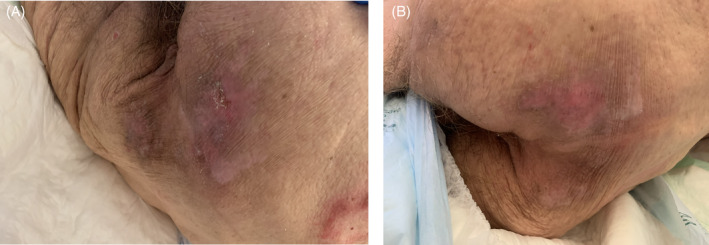
Case 11: Prevention of skin lesions with unique combination of hyaluronic acid and amino acids spray twice a day.

She was treated with thin colloid dressing which resulted in pain and skin damage on removal. Treatment with the combination spray was started twice a day at nappy change (Figure 11A,B).

The combination spray treatment was deemed to be so successful—increases skin respiration in cases of skin damage in incontinence—that in our clinic it is now used as an off‐label preventative measure for skin lesions in elderly patients.

## 5 CASE NOTES (AUTHOR CP)

### 5.1 Case 12: A combination of hyaluronic acid and amino acids in gel/cream formats in the management of necrosis in the heel area after orthopaedic surgery

A 39‐year‐old patient was referred (19/05/2020) by an orthopaedic surgeon in our hospital for dehiscence—necrosis in the heel area after orthopaedic surgery. She was previously treated with betadine gel (povidone‐iodine), sofargen ointment (silver sulfadiazine) and foam. However, her wound did not heal which created problems as she needed to start chemotherapy for abdominal sarcoma as soon as possible.

**FIGURE 12:**
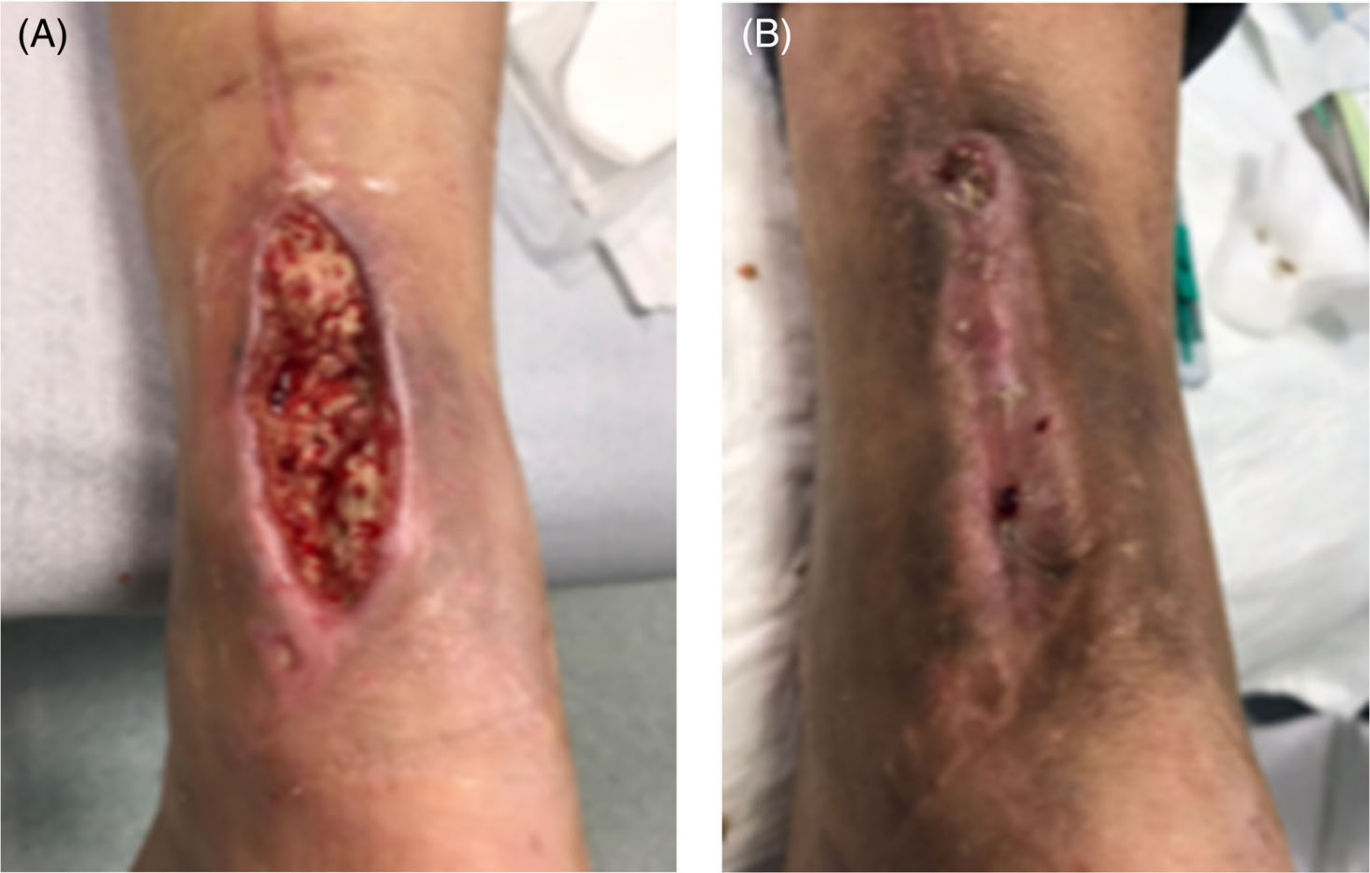
Case 12: Necrosis in heel area after orthopaedic surgery (A) before and (B) after treatment with unique combination of hyaluronic acid and amino acids (gel) (three times a week for 3 months (19/05 to 19/08) followed by Vulnamin cream after debridement as maintenance therapy cream.

Treatment with the combination gel was started—three times a week for 3 months (19/05 to 19/08). After one‐month HA in a solid pad was applied twice a week. The combination of hyaluronic acid and amino acids gel was effective providing debridement and angiogenesis, re‐epithelization and progressive closure. Treatment was well tolerated, and there was no maceration of perilesional skin. The combination cream was used after the combination gel, post debridement as maintenance therapy (Figure 12A,B).

**Take home messages**

A combination of hyaluronic acid and amino acids in cream or gel format is

- Easy to use.
- Possible self‐management.
- Progressive wound area reduction.
- Smooth scar to avoid recurrencies.
- Quick epithelialization.
- Surgical dehiscence effective treatment.
- New ‘off label’ indications.
- Not only epithelialization.
- Treatment for chronic skin damage.
- Scar treatment?

## 6 CLINICAL IMAGE LIBRARY (AUTHOR PC)

**FIGURE 13:**
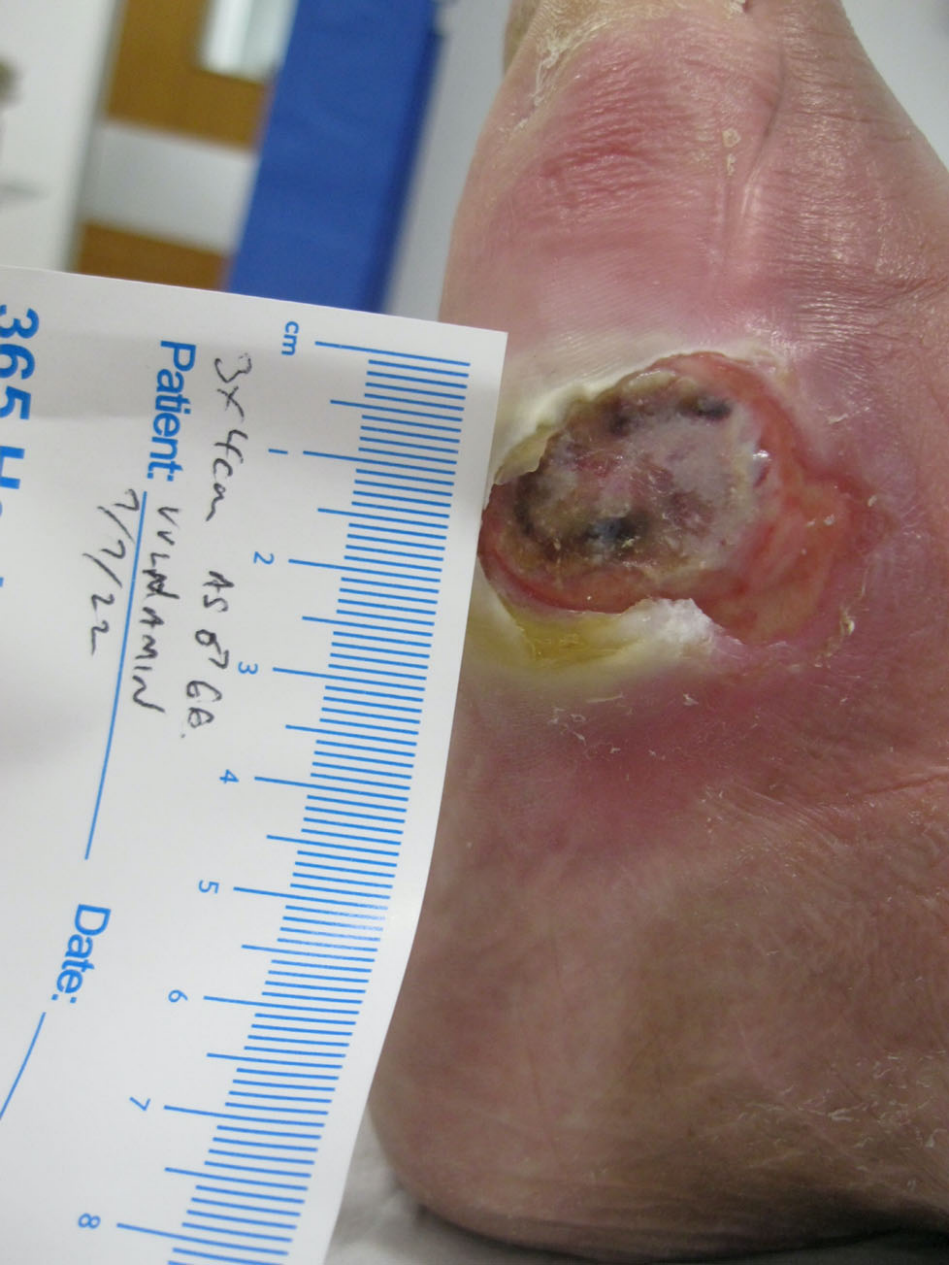
Case 13: 66‐year‐old man, 7 July 2022.

**FIGURE 14:**
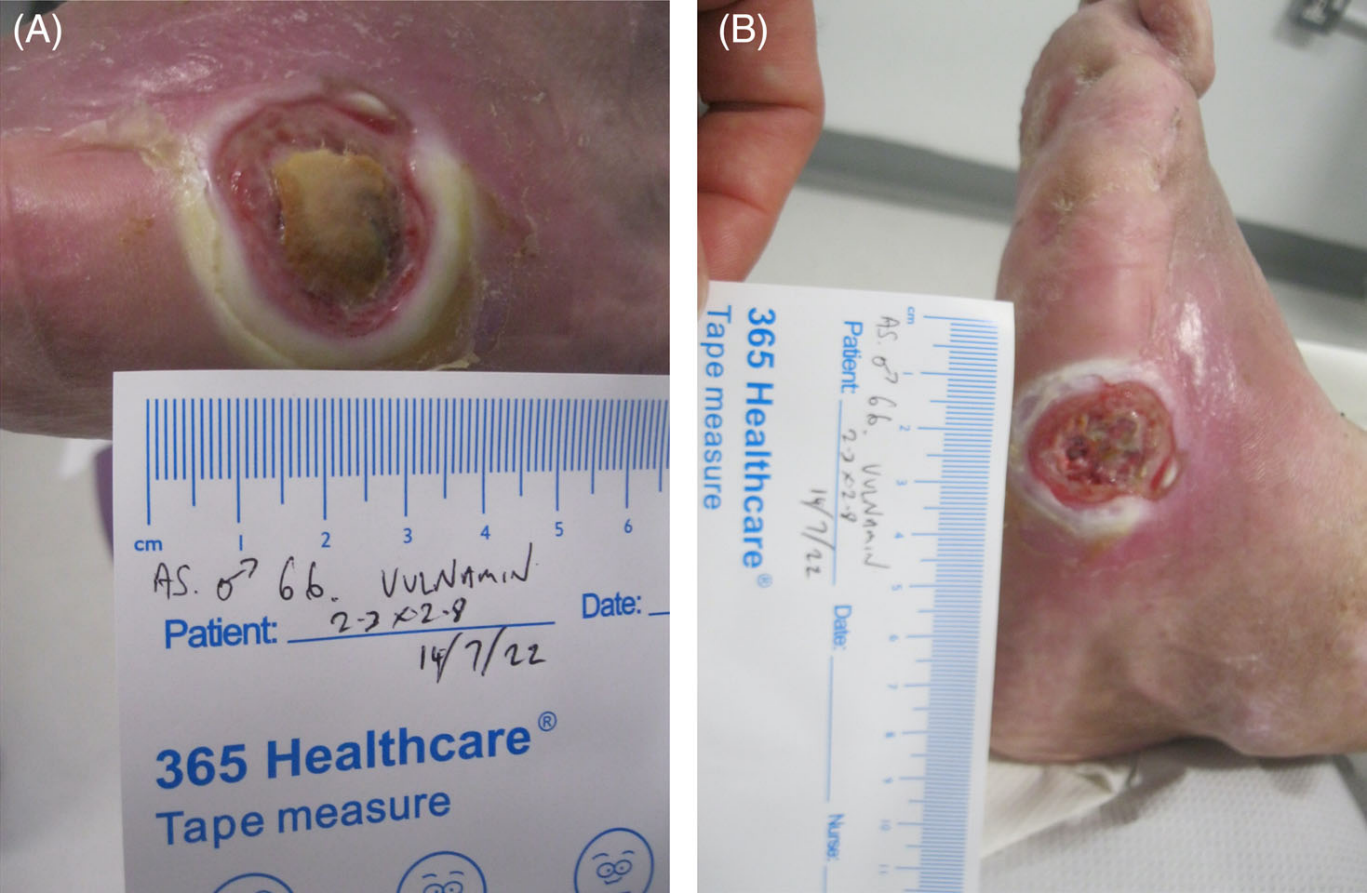
Case 13: 14 July 2022.

**FIGURE 15:**
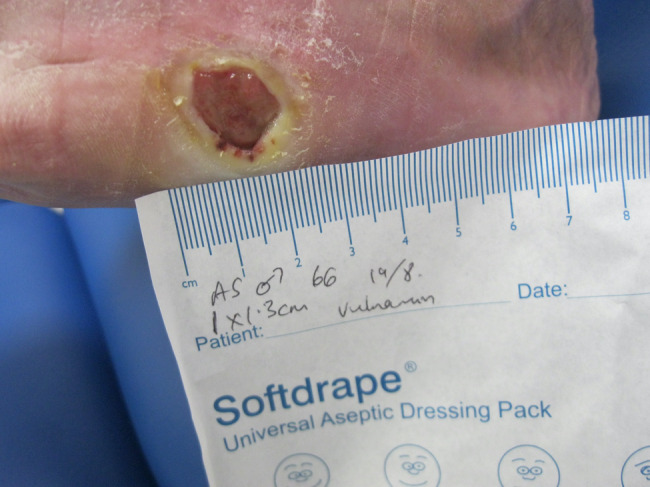
Case 13: 19 July 2022.

**FIGURE 16:**
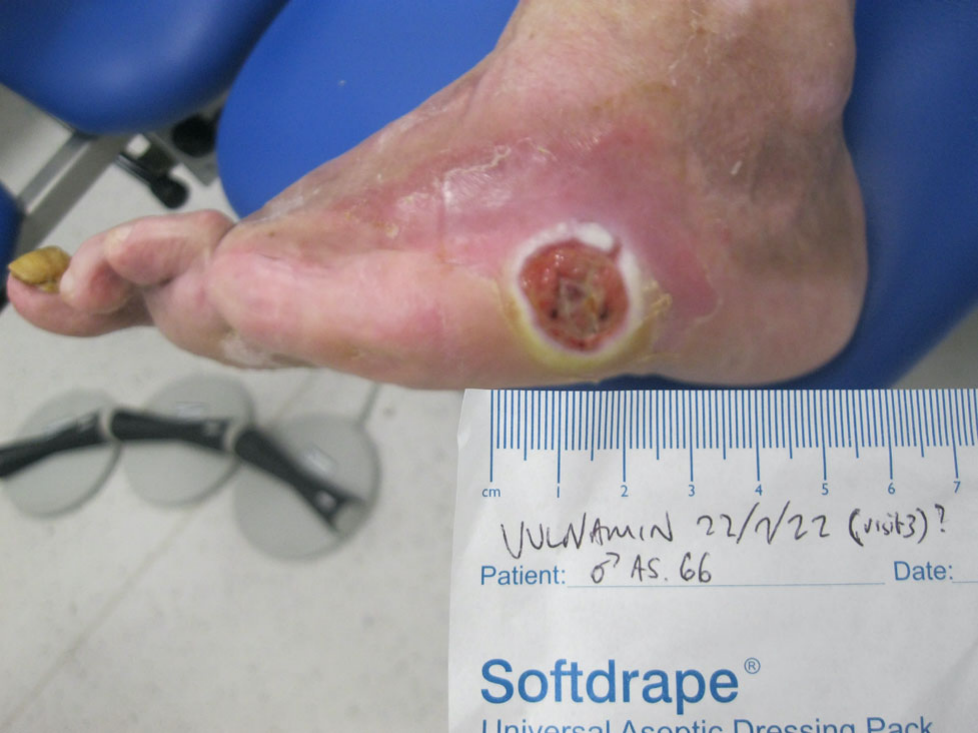
Case 13: 22 July 2022.

**FIGURE 17:**
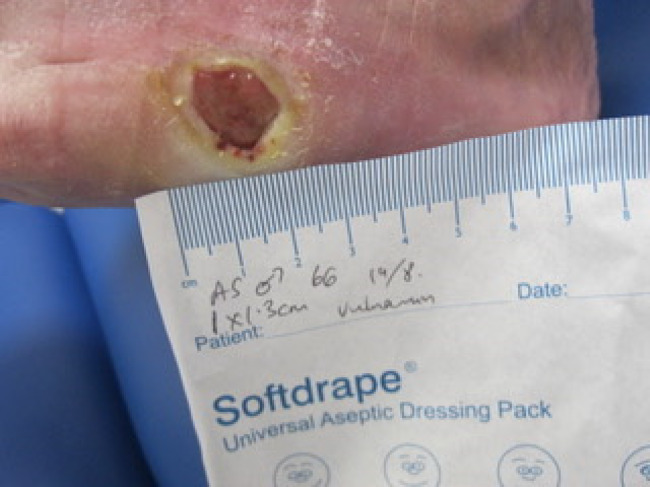
Case 13: 14 August 2022.

A clinical image library involving two patients with complex wounds, successfully treated with a combination of hyaluronic acid and amino acids in gel format was examined and two patients highlighted. The first patient illustrated was a 66‐year‐old male who attended clinic on 16 June 2022 (first visit) with an infected (mild to moderate) wound (no bone involvement) on left lateral styloid process of his foot which had been there for 21 days. The patient had neuropathy and ischemia (coexisting diagnosis of Charcot neuroarthropathy that went on to require right below the knee amputation). His wound was promptly treated with combination gel and Kliniderm foam silicone as a secondary dressing, as necessary. Wound characteristics were monitored regularly (Table 1). The patient's wound healed—length, width, depth and area gradually and significantly decreased, and his wound was completely healed after 10.7 weeks of treatment with combination gel. The reduction in wound area from 9.42 to 0.42 cm^2^ was determined to be ‘remarkable’ given the patient's concomitant conditions. There was a 95.5% overall reduction in wound area over the treatment period (Table 1). The photo gallery demonstrates the progression of the wound over time and the optimal clinical response to the combination gel (Figures 13, 14, 15, 16, 17).

**TABLE 1:** Rapid healing of an acute foot wound in 66‐year‐old male following treatment with a combination of hyaluronic acid and amino acids in gel format.

	Date	Wound size length (cm)	Wound size width (cm)	Wound size depth (cm)	Wound area oval (cm^2^)
Visit 2	07/07/2022	3.0	4.0	0.2	9.42
Visit 3	14/7/2022	2.3	2.8	0.2	5.06
Visit 3	22/07/2022	2.0	2.5	0.1	3.93
Visit 5	29/07/2022	1.9	2.0	0.1	2.98
Visit 6	05/08/2022	1.8	1.9	0.2	2.96
Visit 7	12/08/2022	1.4	1.5	0.2	1.65
Visit 8	19/08/2022	1.0	1.3	0.1	1.02
Visit 9	30/08/2023	1.0	1.3	0.1	1.02
Visit 10	06/09/2022	1.0	1.3	0.1	1.02
Visit 11	13/09/2022	0.3	0.9	0.1	0.21
Visit 12	20/09/2022	0.6	0.9	0.1	0.42
Wound area difference (%)	95.5				
Weeks in evaluation	10.7				

**TABLE 2:** Rapid healing of an acute foot wound in 77‐year‐old male following treatment with a combination of hyaluronic acid and amino acids in gel format.

	Date	Wound size length (cm)	Wound size width (cm)	Wound size depth (cm)	Wound area oval (cm^2^)
Visit 2	28/07/2022	1.7	2.2	0.6	2.94
Visit 3	04/08/2022	1.7	2.2	0.6	2.94
Visit 3	11/08/2022	1.4	1.9	0.2	2.09
Visit 5	18/08/2022	1.0	1.8	0.2	1.41
Visit 6	26/08/2022	0.9	1.6	0.2	1.13
Visit 7	02/09/2022	0.9	1.6	0.2	1.13
Visit 8	09/09/2022	0.5	1.5	0.5	0.59
Visit 9	30/08/2023	1.0	1.3	0.1	1.02
Visit 10	16/09/2022	0.4	1.0	0.5	0.31
Visit 11	23/09/2022	0.7	0.8	0.4	0.44
Visit 12	30/09/2022	0.4	1.0	0.3	0.31
Visit 13	14/10/2022	0.4	1.0	0.3	0.31
Wound area difference	89.0%				
Weeks in evaluation	11.4				
Wound healed	04/12/2022				

**FIGURE 18:**
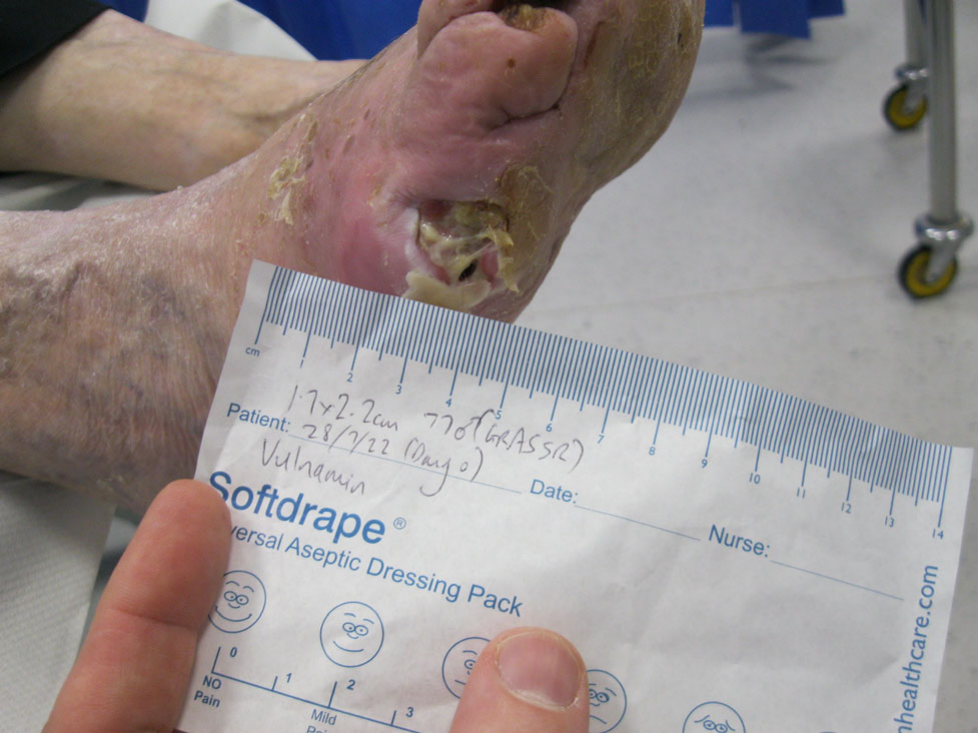
Case 14: 77‐year‐old man, 28 July 2022.

**FIGURE 19:**
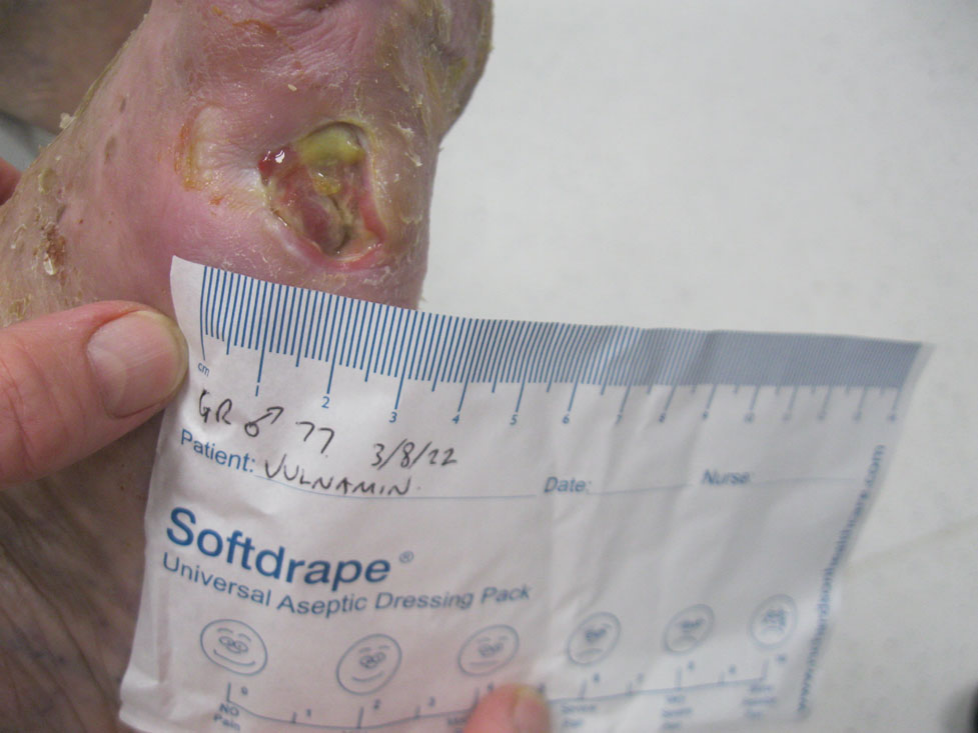
Case 14: 3 August 2022.

**FIGURE 20:**
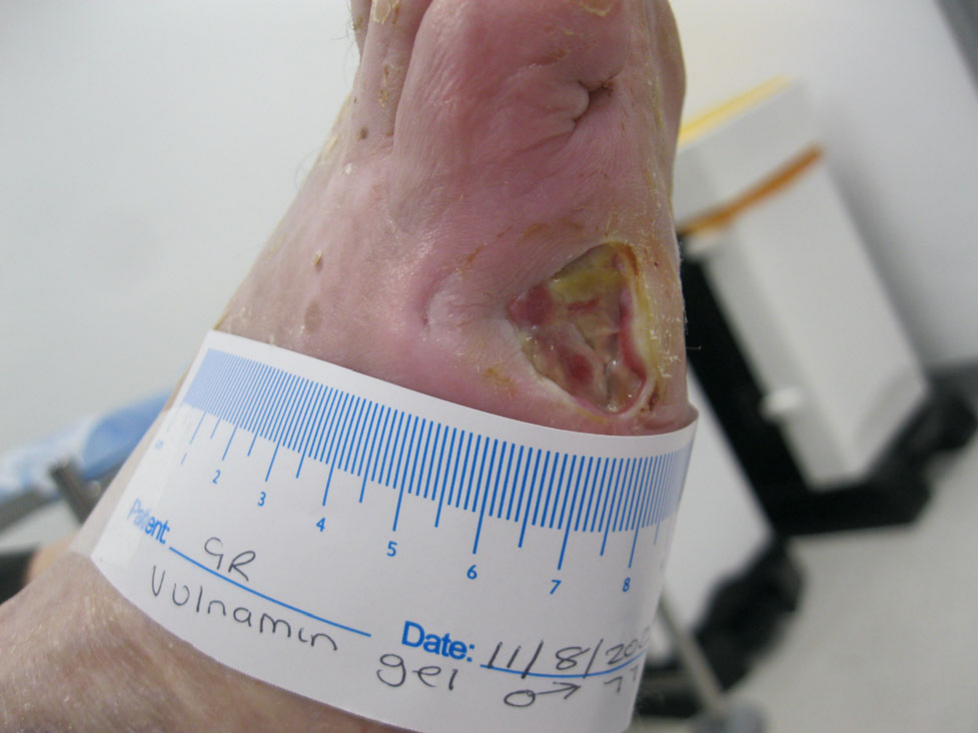
Case 14: 11 August 2022.

**FIGURE 21:**
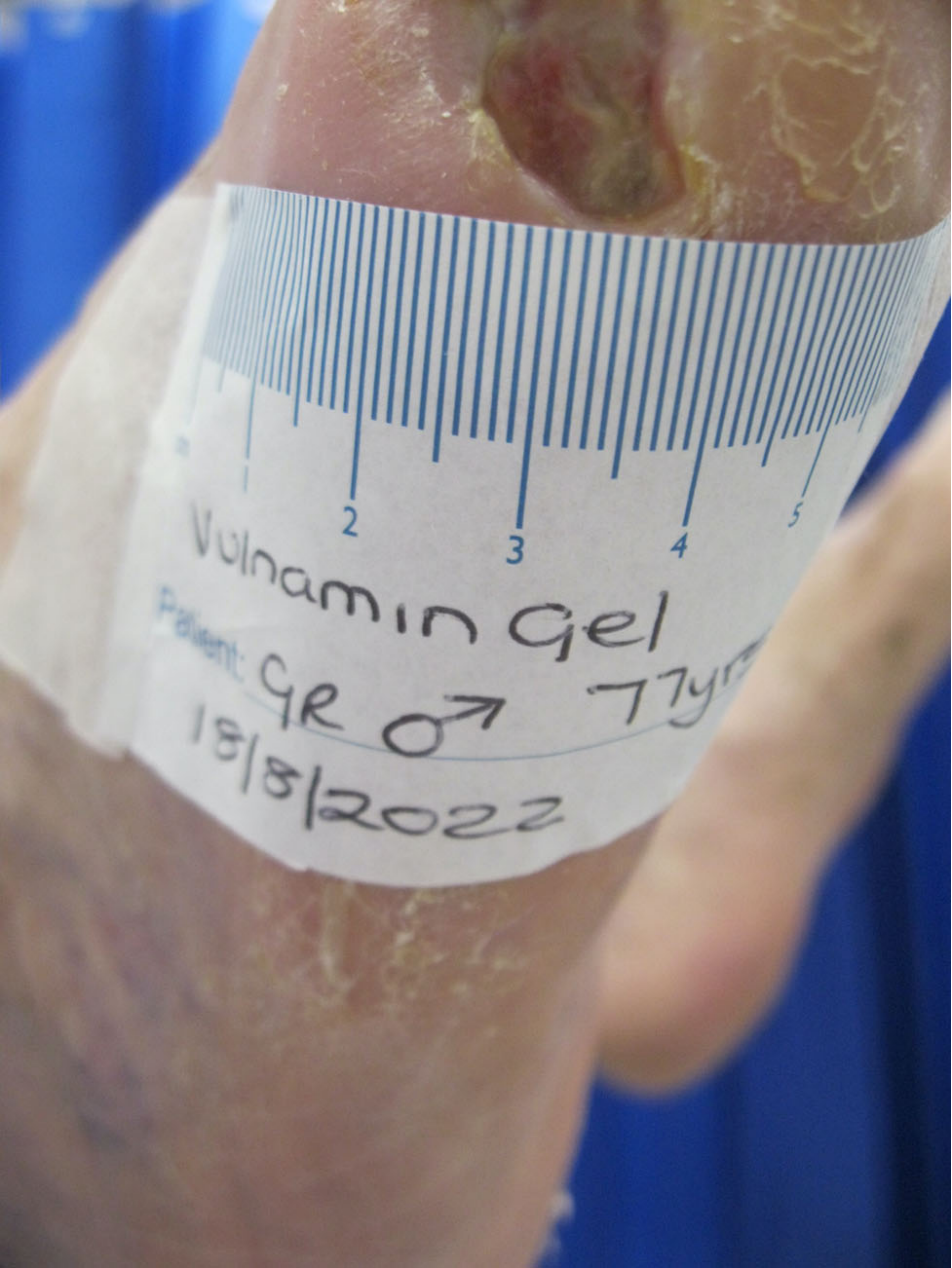
Case 14: 18 August 2022.

**FIGURE 22:**
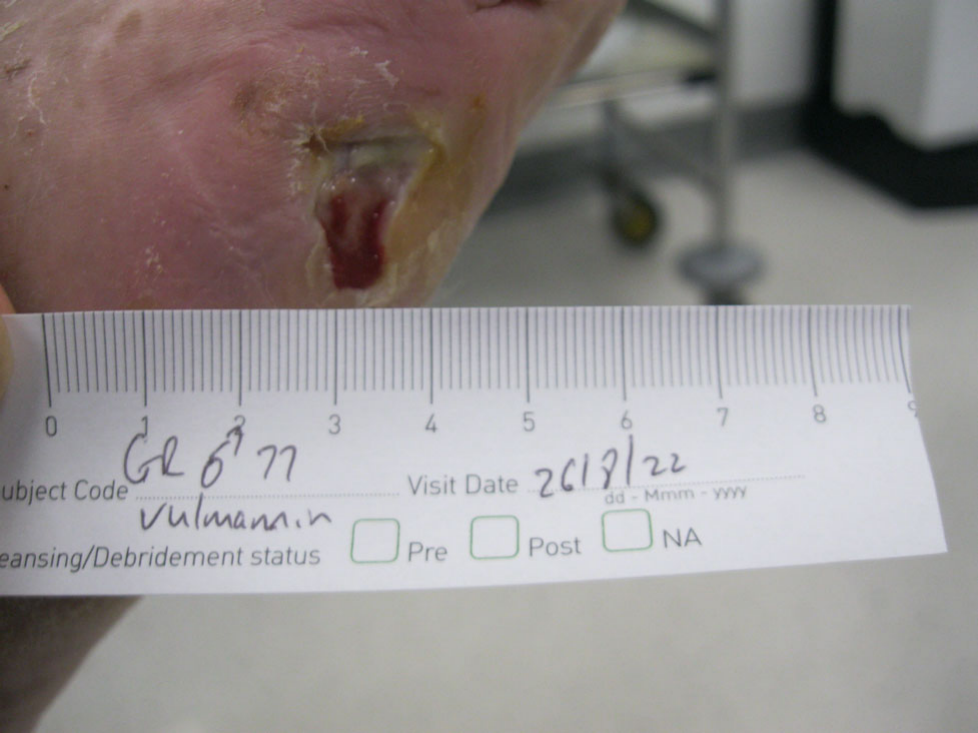
Case 14: 26 August 2022.

**FIGURE 23:**
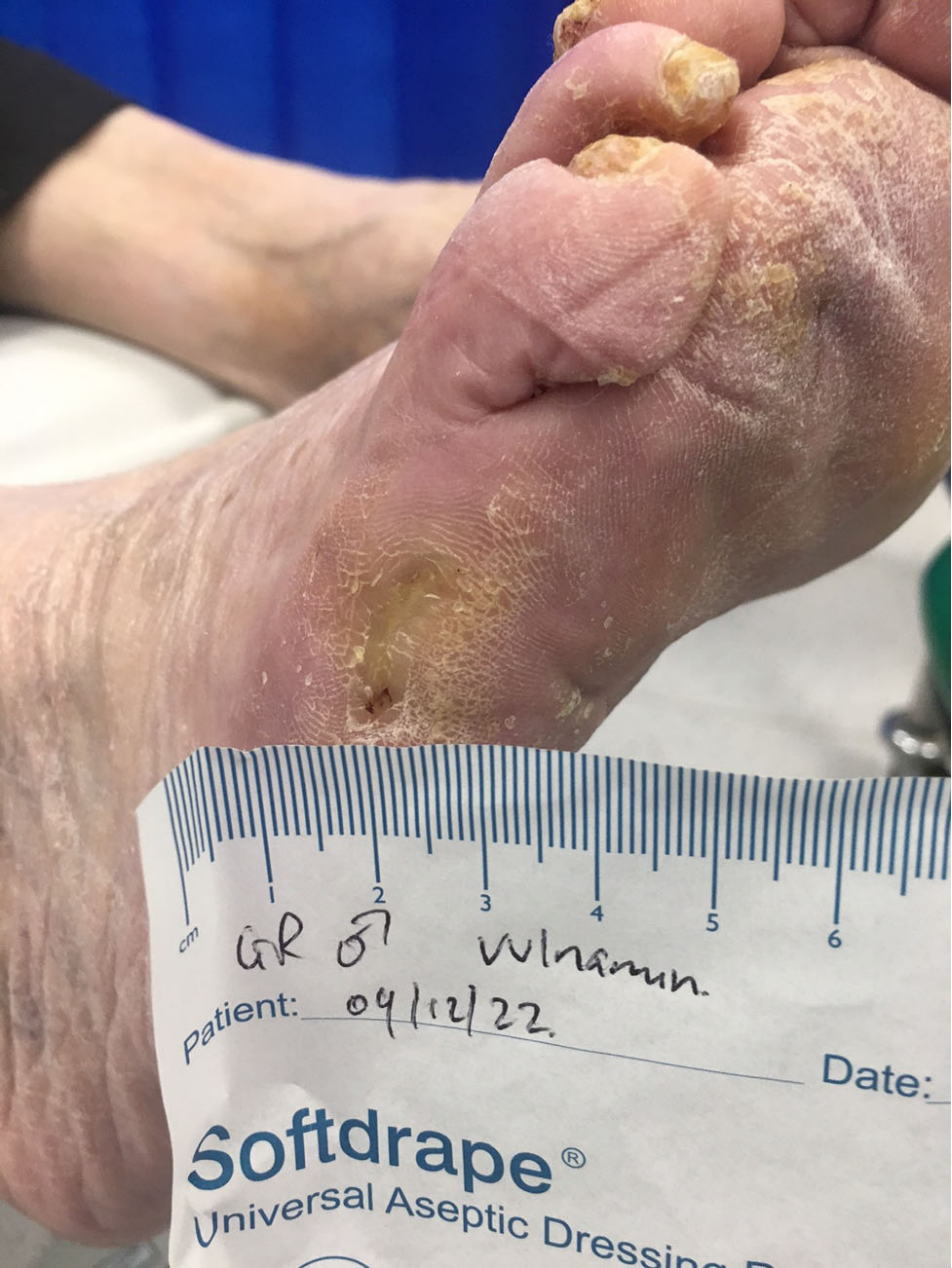
Case 14: Healed, 4 December 2022.

The second patient illustrated was a 77‐year‐old male who attended clinic on 15 June 2022 (first visit). He had had a chronic wound at the base of his right fifth metatarsal for 43 days. He was also neuropathic and had ischemia. On observation, the depth of the wound was to the bone and was infected osteomyelitis – OM. Likewise, his wound was treated with the combination gel and Kliniderm foam silicone as a secondary dressing, as necessary. Wound characteristics were monitored regularly (Table 2). The patient's wound healed—length, width, depth and area gradually and significantly decreased. Importantly the depth of his wound reduced markedly with treatment. His wound was completely healed after 11.4 weeks of treatment with the combination gel. Wound size (length) was reduced fourfold, and there overall was an 89.0% reduction in wound area with treatment (Table 2). The photo gallery demonstrates the progression of the wound over time, and the optimal clinical response to the combination gel (Figures 18, 19, 20, 21, 22, 23).

### 6.1 Expert panel consensus: Place of unique combination of hyaluronic acid and amino acids in wound management

The consensus of the expert panel was that there was a place for a unique combination of hyaluronic acid and amino acids product within wound management. The panel answered a number of questions and considered the evidence to support the technology, and the case studies presented to arrive at consensus on the questions below.

The real‐world clinical experience on the efficacy of these products in patients with significantly different wound types allowed the panel to draw‐up guidelines for clinicians who have not had first‐hand experience of the product in their clinical practice. The panel made recommendations on the optimal wound management strategies with the combination of hyaluronic acid and amino acids in various formats—from type of wounds and goals of treatment to QoL issues and when, where and how the combination product should be used. The combination of hyaluronic acid and amino acids range has been shown to be effective in both chronic and acute wounds (Table 3).

**TABLE 3:** A combination of hyaluronic acid and amino acids products effective in both chronic and acute wounds.

Chronic	Acute
Venous leg ulcers	Epidermolysis bullosa
Pressure ulcers	Burns (second degree)
Diabetic foot ulcers	Wound adhesions/wound separation dehiscence
Arterial ischemic wounds	Skin tears
Inflammatory	
Hidradenitis suppurativa (wound care where there no current guidelines)	

#### 6.1.1 How often should the combination of hyaluronic acid and amino acids be used?

In general, the panel felt that the combination product should be used twice a week when dressings are changed and/or when the patient attends clinic. Practicality and methods employed by individual clinics/clinicians play a major role in frequency of use. Factors influencing the frequency of use include the workload of the team and the type of wound being treated. For example, a highly exudative wound may require more frequent applications, also the type of formulation used plays an important role. The powder format may be used less frequently compared with the cream, but it also depends on the characteristics of the individual wound and to a certain extent the specific needs of individual patients.

#### 6.1.2 When should the combination of hyaluronic acid and amino acids products be used?

This depends on several factors including health economic data—need to demonstrate cost‐effectiveness to recommend use at the outset/first patient visit. Importantly, products in the range can be used synergistically, and there is room for overlap.

#### 6.1.3 Where does the combination products act in the wound healing process?

The expert panel highlighted that the combination of hyaluronic acid and amino acids products act in the three phases of tissue repair, namely, coagulation, inflammation and remodelling (Table 4).

**TABLE 4:** The combination of hyaluronic acid and amino acids products in the three phases of tissue repair.

Phase	Evidence
Coagulation	In vivo
Inflammation	In vitro, in vivo—cytokines, increase in vascular factors, hyperpigmentation, scar, biopsy before/after and during, decreased in jaw necropsy
Remodelling	In vivo—not an antimicrobial need to ensure good control, maybe usual in local infection as TGF to wound also the combination powder may act as a barrier to secondary infection, silver dressing may be reached if too much powder applied—nothing can penetrate.

#### 6.1.4 How should the combination of hyaluronic acid and amino acids product line be categorized?

As the combination of hyaluronic acid and amino acids is a novel product with a unique combination of active ingredients and effects on wounds, it does not fall into one specific product category. The expert panel considered that it could be categorized as one of the following:

1. Tissue feeder
2. Debrider
3. Nourishing
4. Facilitator/regeneration
5. Tissue regenerator

#### 6.1.5 How should the combination of hyaluronic acid and amino acids range of products be used in different wound types?

The panel reiterated that it is important to select the most appropriate product according to the characteristics of the wound to be treated. For example, the combination powder is the recommended option for high exudate wounds while gel is more appropriate for deep wounds (Table 5).

**TABLE 5:** Recommendations for use of the combination of hyaluronic acid and amino acids product range according to wound type.

	Exudate	Depth (staging of type of wound)	Wound size	Ease of application
Powder	+++	++	+++	+
Cream	+	++	++	++
Gel	+	+++	++	++
Spray (prevention)	0	+	+++	+++

#### 6.1.6 Can the combination of hyaluronic acid and amino acids products be used simultaneously?

The panel agreed that the products can be combined depending on wound/patient characteristics. For example, one could use the powder plus a thin layer of cream, and it is also possible to combine with a foam dressing to control exudate. The bottom line comes down to costs. Combining two of the combination products can be expensive. However, if the efficacy improvements are obtained, it may be a cost‐effective approach. An added advantage of the product range is that it also allows flexible usage. For example, one can move from one product to the other (e.g., start with powder in highly exudative wounds and then move on to cream as exudate subsides).

#### 6.1.7 What secondary dressings should be used with the combination products?

Secondary dressings are important in effective wound management—in addition to their impact on the efficacy of the primary dressing, they protect, reinforce and cover the primary dressing. Alginate dressings have good exudate absorption properties (they form a gel after binding to wound exudate) and thus can be used in wounds with a large amount of exudate. Foam dressings are suitable for handling moderate‐to‐high volumes of wound exudate, and they provide thermal insulation and maintain moisture to the wound and prevent damage on removal. The combination products are suitable for use with these dressings according to the type of wound (Table 6).

**TABLE 6:** Recommendations for use of the product range according to wound type.

	Exudate amount +	Exudate amount (medium) ++	Exudate amount (heavy) +++
Powder	Contact layer	Alginate hydrofibre	Foam and alginate hydrofibre
Cream	Contact layer	Alginate hydrofibre	Foam and alginate hydrofibre
Gel	Contact layer	Alginate hydrofibre	Foam and alginate hydrofibre

#### 6.1.8 When should use of the combination products be avoided?

The combination of hyaluronic acid and amino acids technology can be used in a wide range of infections, and there are no contraindications as such, to their use but they should not be used in wounds with overt infection. Secondary antiseptic dressings are recommended in these situations. They should be used under medical supervision and should not be used in subjects with known allergy to product/ingredients.

## 7 CONCLUSIONS AND FUTURE PERSPECTIVES

We have seen that with increasing numbers of elderly patients, there is a concurrent increase in the incidence of both chronic and acute wounds due to conditions such as diabetes and chronic vascular diseases. There is a large unmet medical need in the effective management of wounds. Delayed treatment and poor healing of wounds not only reduce the QoL of patients but cause a heavy medical and financial burden for healthcare systems worldwide. Optimizing wound management to prevent infection, accelerate wound healing and reduce patients' suffering and improve cost‐effectiveness is therefore paramount. The selection of the most appropriate modern dressing product is a challenge for clinicians. The characteristics of the ‘ideal’ dressing include maintaining moisture balance, promoting oxygen exchange, stimulating growth factors, facilitating autolytic debridement and promoting the production of granulation tissue and re‐epithelialization.^7^

Wound healing is a dynamic process made up of distinct phases, and thus the effective management of wounds requires that dressings provide the appropriate environment at each phase of the healing process. In vitro and in vivo trials with the combination products with their combination of hyaluronic acid and amino acids, act at all stages of the wound healing process, induce and promote re‐epithelialization of wounds and are associated with a marked reduction in inflammatory infiltrate and colonization of the lesion by newly formed vessels. In cutaneous wounds, topical combination of hyaluronic acid and amino acids modulates the inflammatory response and stimulates activation and proliferation of fibroblasts with a rapid and significant increase in the percentage of wound area filled with collagen fibres, producing a collagen fibre network and subsequently reducing healing time. The clinical cases presented by the expert panel provide additional evidence that the unique combination of hyaluronic acid and amino acids within a range of formulations comes close to providing the ideal characteristics necessary for effective wound healing with the possible exception of wounds with overt infection where secondary antibacterial dressing are recommended.

Looking to the future, large‐scale, randomized, controlled clinical trials are required in different clinical settings such as nursing homes as a significant proportion of wound healing occurs in the out‐of‐hospital setting. In addition, we need more health economic data on costs of treating the different type of wounds, length of treatment recommended for use, combinations with other dressings and regulations/rules for reimbursement in different countries.

## FUNDING INFORMATION

This research did not receive any specific grant from funding agencies in the public, commercial, or not‐for‐profit sectors. Medical writing support was provided by Edra S.p.A., with an educational grant from Professional Dietetics S.p.A., www.professionaldietetics.com.

## CONFLICT OF INTEREST STATEMENT

The authors report no conflicts of interest.

## Data Availability

The data that support the findings of this study are available from the corresponding author upon reasonable request.
